# Alternative Agents to Colcemid for Obtaining High-Quality Metaphase Spreads

**DOI:** 10.3390/ani15101476

**Published:** 2025-05-20

**Authors:** Michele Zannotti, Marco Battelli, Pietro Parma

**Affiliations:** Department of Agricultural and Environmental Sciences, University of Milan, Via Celoria 2, 20133 Milano, Italy; michele.zannotti@unimi.it (M.Z.); marco.battelli@unimi.it (M.B.)

**Keywords:** FISH, metaphase, cattle, colcemid

## Abstract

In cytogenetics, the ability to perform FISH (Fluorescence In Situ Hybridization) experiments using probes that map very closely together depends on the capacity to produce sufficiently long chromosomes. Traditionally, colcemid is the chemical agent used to obtain metaphase spreads. However, various substances have been reported to arrest cells in an earlier stage of mitosis than the metaphase, potentially providing longer chromosomes. In this study, we tested seven substances different from colcemid, which, according to the literature, have this capability: Vinblastine, Combretastatin A-4, Podophyllotoxin, Org9935, Nocodazole, Paclitaxel, and Griseofulvin. All substances were tested on lymphocyte cultures derived from whole blood at the same concentration: 0.1 µg/mL. Among these, Org9935 and Griseofulvin were confirmed to have the ability to produce metaphases with longer chromosomes compared to those obtained with colcemid.

## Simple Summary

This study analyzes the effectiveness of different substances in producing longer chromosomes for FISH experiments in cytogenetics. Traditionally, colcemid is used, but researchers tested seven alternatives, demonstrating that some are more effective. In particular, Org9935 and Griseofulvin proved to be the best at generating longer chromosomes.

## 1. Introduction

In the field of cytogenetic analysis, FISH technology represents a fundamental investigative tool. Initially created to map genomic elements on metaphase chromosomes, it has subsequently also been used for chromosome identification, characterization of cytogenetic abnormalities, and comparative studies between different species [1].

Among zoo-economic animals, several species have attracted significant interest from cytogeneticists, mostly as a result of the identification of chromosomal abnormalities that impact fertility. Examples include the discovery of the Robertsonian translocation 1;29 in cattle [2] the identification of various reciprocal translocations in pigs [3], or the impact of monosomies or trisomies of sex chromosomes in horses [4].

The possibility of obtaining good results with FISH essentially depends on two factors: having probe material available and having metaphase plates with sufficiently elongated chromosomes to observe probes close to each other distinctly. The first condition was solved with the production of BAC libraries by two research institutes: CHORI (CH240; https://bacpacresources.org/, accessed on 15 May 2023) and INRA (btINRA, https://abridge.inrae.fr/en/, accessed on 15 May 2023). Clones belonging to these two libraries are easily identifiable through the most common genome assembly display sites. The availability of elongated metaphases depends on when the cells in mitosis are arrested and thus the chromosomes are displayed. Although 60 years have passed since the discovery of Rob1;29, the procedure used to produce metaphases has not changed much—metaphase chromosomes are still obtained today through the action of colcemid [5]. During cell division, colcemid (also known as demecolcine) inhibits mitosis at the metaphase by inhibiting spindle formation. In addition to this compound, there are several other substances, often used as anti-cancer drugs, that can arrest the mitosis process. Moreover, some of these can arrest this process at times prior to metaphase and thus potentially produce longer chromosomes.

The aim of this work was to test whether one or more of these substances can provide less condensed bovine chromosomes than those normally obtained using colcemid.

## 2. Materials and Methods

### 2.1. Substances Considered

Seven compounds were used in this trial in addition to colcemid, which was used as a reference. The compounds were identified after research aimed at identifying substances capable of arresting mitosis at a stage prior to the metaphase. These substances, belonging to the class of polymerization or depolymerization inhibitors, are listed in Table 1.

### 2.2. Cell Cultures from Peripheral Blood

Cell cultures were produced according to the current protocol [3]. The eight cultures were made from a super-mix prepared containing 32 mL of RPMI 1640 (with added antibacterials and antimycotics), 8 mL FCS, and 4 mL whole blood obtained from one female subject. Finally, 660 µg of concanavalin A (15 µg/mL final concentration) was added. This mix was then divided into 8 independent cultures and placed at 37.5 °C for 72 h. All substances used for mitosis arrest, including colcemid, were aliquoted at 100 µg/mL. The tested substances were added to a final concentration of 0.1 µg/mL 90 min before the cells were harvested. The cultures were then treated as usual [3] and, finally, one slide for each treatment was prepared and Giemsa stained. The two most promising compounds identified after the first experiment (Griseofulvin and Org9935) were tested in a second experiment (together with colcemid), which was identical to the first except for the exposure time to the agent used to arrest metaphases: 120 min.

### 2.3. Measurement of Chromosomes and Mitotic Index

The mitotic index is expressed by the percentage of cells that, at a defined time and location, are in mitosis. To obtain the indices for the different tested substances, it was necessary to determine for each slide the number of cells fixed in mitosis and that of total cells and then to relate them. This was achieved by observing the metaphase plates at 20× magnification and recording the numbers of total and metaphase cells with a cell counter. Given the objective of identifying which substances result in less condensed chromosomes than those treated with colcemid for the same exposure time, the average length of the chromosomes obtained from each treatment was measured.

To make a valid comparison, it is necessary to measure the same chromosome for each metaphase and slide. This necessity makes BTAX the chromosome of choice for measurements because it is the only one clearly identifiable during the observation of a Giemsa-stained metaphase. Moreover, considering that the size of an X chromosome can be affected by the process of X inactivation, both X chromosomes were measured for each metaphase. BTAX images were obtained by taking photographs, with a camera connected to the PC, of 50 metaphases (45 for the second experiment) on each slide, framed one by one at 100× magnification. To avoid invalidating the results of the investigation, the enlarged and photographed metaphases were randomly chosen from those on each slide and were not selected according to the appearance of their chromosomes. To perform the measurement, images of the metaphases were transferred to PowerPoint pages grouped by slide number. Using the program’s tools, X chromosomes were cropped from each image and enlarged all by the same percentage, locking the proportions. The pages were printed, and the measurements were carried out.

### 2.4. Statistical Analysis

The statistical analysis of the length of BTAX chromosomes was performed with the MIXED procedure of SAS 9.4 (SAS Institute Inc., Cary, NC, USA), with the plate as a random variable, using the following model:

Yij = µ + Ti + Pj(T) + εij,

where Yij is the length of BTAX chromosomes, μ is the overall mean, Ti is the fixed effect of the treatment (i = 1, …, 8), Pj(T) is the random effect of plate j within the treatment, with j = 1, …, 50, and εij is the residual error.

For the second experiment, the effect of exposure time was evaluated separately for each of the three compounds using the MIXED procedure with the following model:

Ykl = µ + Ek + Pl(E) + εkl,

where Ykl is the length of BTAX chromosomes, μ is the overall mean, Ek is the fixed effect of the exposure time (i = 90 or 120 min), Pl(E) is the random effect of the plate j within the exposure time, with j = 1, …50, and εkl is the residual error.

Differences between the least square means were evaluated using Tukey’s method for comparison.

The equality of variances in BTAX length measurements was carried out by means of Levene’s test. Finally, the comparison between the different mitotic indices was made with the chi-squared test, and for correlations, the Spearman rank correlation coefficient was calculated.

## 3. Results

BTAX measurements (in mm) of the 50 observed metaphases provided the results shown in Table 2.

According to the results, there is a statistically significant difference (*p* < 0.05), and certain drugs can actually result in metaphasic or pro-metaphasic plates with longer chromosomes. For this parameter, the most promising substance seems to be Org9935, which is able to produce longer chromosomes with less variability than other compounds.

**Figure 1:**
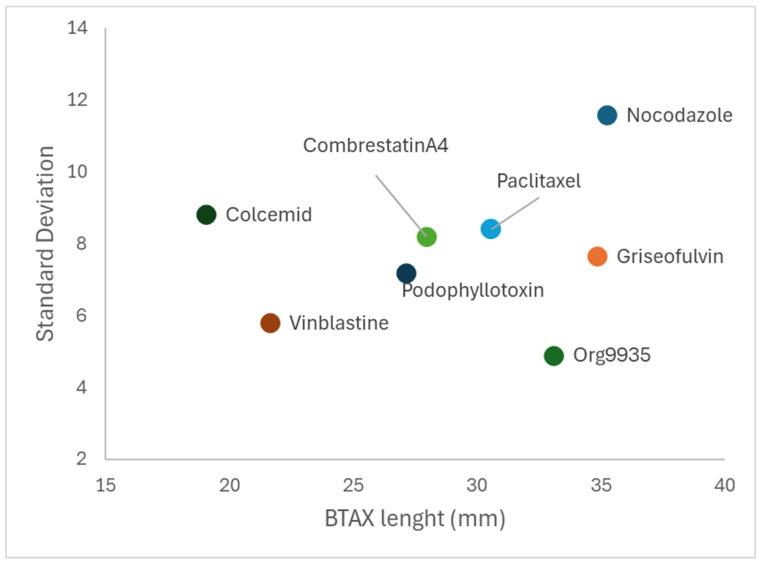
Relationship between BTAX measurement (mm) and measurement variability (expressed as standard deviation) across treatments.

The variability of the data obtained also showed a significant difference with respect to the length of BTAX, and the two variables showed a Spearman rank correlation coefficient of 0.5952 (Figure 1). This value, while indicating a trend, does not reach the threshold to be significant for *p* < 0.05. An example of metaphases obtained in the various treatments is given in the Supplementary Information S2.

**Figure 2:**
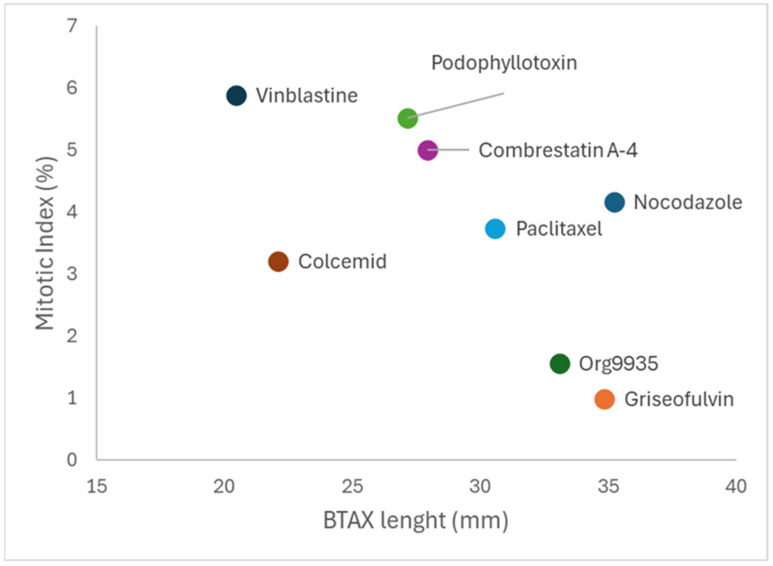
Relationship between BTAX measurement (mm) and mitotic index (expressed in %) in the various treatments.

The results concerning the mitotic index values are shown in Table 3. Additionally, in this case, a statistically significant difference (*p* < 0.05) was observed for the values obtained. A correlation analysis between chromosome length and mitotic index (Figure 2) showed that the value of the Spearman rank correlation coefficient is −0.5714, a value that, even if it indicates a trend, does not reach the significance threshold for *p* < 0.05.

It is a known fact that when using colcemid, an increase in the mitotic index can be achieved by increasing the exposure time of cell cultures to this compound. However, this has a negative consequence—the shortening of chromosomes, which could affect the identification of certain types of abnormalities [14]. To test whether these consequences are present in the most promising substances tested, we repeated the experiments considering only Org9935, Griseofulvin, and colcemid and left the cell cultures in exposure for 120 min instead of the 90 min previously used. We excluded Nocodozole from this analysis because, although it showed excellent results in terms of chromosome elongation, it showed a very wide variability. The results obtained are shown in Table 4.

These results, in addition to confirming what was previously observed about the production of longer chromosomes, show that prolonged exposure also results in a shortening of chromosomes for these two substances.

## 4. Discussion

The aim of this experiment was to explore the possibility of identifying alternative substances to colcemid for halting mitotic division before the metaphase. The ultimate goal is to obtain elongated chromosomes to be used in high-resolution FISH experiments. The seven compounds tested (plus colcemid as the standard reference) were identified through a literature review. From this perspective, we do not exclude the existence of other substances capable of yielding equal or better results than those obtained. After a preliminary experiment, three substances produced statistically similar results: Nocodazole, Griseofulvin, and Org9935. These three compounds generated chromosomes that were on average 55% longer than those obtained with colcemid. However, Nocodazole showed a high degree of variability, which led us to exclude it from the second round of testing. We believe that obtaining metaphases with highly variable chromosome lengths is not useful. It is well-known that extending the exposure time of metaphases to colcemid increases the mitotic index but also causes a progressive shortening of the chromosomes. The second experiment aimed to verify whether Nocodazole and Org9935 exhibited the same behavior. Results showed that prolonged exposure to these two substances also caused a reduction in chromosome length. However, this reduction was less pronounced compared to what was observed with colcemid: 18% vs. 39%. This is an interesting result because it suggests that the exposure time can be increased without significantly compromising chromosome length. The use of agents alternative to colcemid for mitotic arrest has been reported in various studies, but to our knowledge, never in whole-blood cultures from cattle. Such experiments often involve the use of cancer cell lines [15]. Among the tested substances was Paclitaxel, which we evaluated and which is considered highly effective in breast cancer treatment [16].

## 5. Conclusions

In conclusion, the experiments performed confirm the possibility of using alternative chemicals—in particular, Griseofulvin and Org9955—for chromosome production in FISH experiments when two or more probes are spaced a few Mb apart.

## Figures and Tables

**Table 1:** Tested substances.

Substance	Action	Reference
Colcemid	P.I.	[6]
Vinblastine	P.I.	[7]
Combrestatin A-4	P.I.	[8]
Podophyllotoxin	P.I.	[9]
Org9935	P.I.	[10]
Nocodazole	P.I.	[11]
Paclitaxel	D.I.	[12]
Griseofulvin	D.I.	[13]

P.I.: polymerization inhibitor. D.I.: depolymerization inhibitor. Manufacturer and catalog number are reported in Supplementary Information S1.

**Table 2:** Lengths of BTAX chromosomes (90 min treatment).

Substance	Mn (mm)	S.D.	C.I.
Nocodazole	35.2 ^a^	11.57 ^a^	31.93–38.51
Griseofulvin	34.84 ^ab^	7.65 ^a^	32.67–37.01
Org9935	33.10 ^ab^	4.89 ^a^	31.71–34.49
Paclitaxel	30.56 ^bc^	8.40 ^ab^	28.17–32.95
Combrestatin A-4	27.94 ^c^	8.21 ^b^	25.61–30.27
Podophyllotoxin	27.14 ^c^	7.17 ^b^	21.10–29.18
Vinblastine	20.46 ^d^	4.95 ^c^	19.05–21.87
Colcemid	22.10 ^d^	4.67 ^c^	20.77–23.43

Mn: mean BTAX length. ^a,b,c,d^: different letters in the column correspond to different least squares means after Tukey’s adjustment (*p* < 0.05). S.D.: standard deviation. ^a,b,c^: different letters indicate statistical significance at *p* < 0.05. C.I.: 95% confidence interval of the mean.

**Table 3:** Mitotic index.

Substance	Cells (n)	Metaphases (n)	Mitotic Index (%)	C.I. 95%
Nocadozole	3681	160	4.17 ^a^	3.53–4.80
Griseofulvin	2227	22	0.98 ^b^	0.57–1.38
Org9935	3429	54	1.55 ^b^	1.14–1.96
Paclitaxel	721	28	3.74 ^a^	2.38–5.10
Combrestatin A-4	1425	75	5.00 ^a^	3.90–6.10
Podophyllotoxin	651	38	5.52 ^a^	3.81–7.22
Vinblastine	848	53	5.88 ^a^	4.35–7.42
Colcemid	1669	55	3.21 ^a^	3.37–4.04

^a,b^: different letters indicate statistical significance at *p* < 0.05. C.I.: 95% confidence interval of the mitotic index.

**Table 4:** Lengths of BTAX chromosomes (120 min vs. 90 min treatment).

Substance	Minutes	Mn	S.D.	C.I.
Griseofulvin	90	34.84 ^a^	7.65	32.67–37.01
Griseofulvin	120	28.09 ^b^	6.64	25.08–30.08
Org9935	90	33.10 ^a^	4.89	31.71–34.49
Org9935	120	27.04 ^b^	5.72	24.18–28.76
Colcemid	90	22.10 ^a^	4.67	20.77–23.43
Colcemid	120	13.31 ^b^	3.94	10.71–14.49

Mn: mean BTAX length: ^a,b^: different letters indicate statistical significance at *p* < 0.05. The significant difference refers to the difference between the two treatments (90′ vs. 120′) for each individual substance, not between individual substances. S.D.: standard deviation. C.I.: 95% confidence interval of the mean.

## Data Availability

The data presented in this study are available upon request from the corresponding author.

## Supplementary Materials

The following supporting information can be downloaded at https://www.mdpi.com/article/10.3390/ani15101476/s1, Supplementary Information S1: Manufacturer and catalogue number for used substance; Supplementary Information S2: representative metaphases.

## Abbreviations

The following abbreviations are used in this manuscript:

FISH	Fluorescence In Situ Hybridization
BAC	Bacterial Artificial Chromosome
CHORI	Children’s Hospital Oakland Research Institute
INRA	Institut national de la recherche agronomique
BTAX	*Bos taurus* X chromosome
