# Supplementary material for: Alternative Agents to Colcemid for Obtaining High-Quality Metaphase Spreads
Source: Animals (Basel). 2025 May 20;15(10):1476. doi: 10.3390/ani15101476 (PMC12108235; doi:10.3390/ani15101476)
Supplement: Supplementary file 1 [file animals-15-01476-s001.zip › animals-3566408-supplementary.pdf]

Alternative Agents to Colcemid for Obtaining High-Quality Metaphase Spreads

Supplementary information S1: Manufacturer and catalogue number for used substance.

Substance	Manufacturer	Catalog number
Colcemide	Sigma Aldrich	C9754
Vinblastine	Sigma Aldrich	V1377
Combrestatin A-4	Sigma Aldrich	C7744
Podophyllotoxin	Sigma Aldrich	P4405
Org9935	Gift of Prof. Jeffrey Jensen from Oregon Health & Science University	
Nocodazole	Sigma Aldrich	M1404
Paclitaxel	Sigma Aldrich	T9171
Griseofulvin	Sigma Aldrich	G4753

Supplementary information *S*2: representative metaphases.

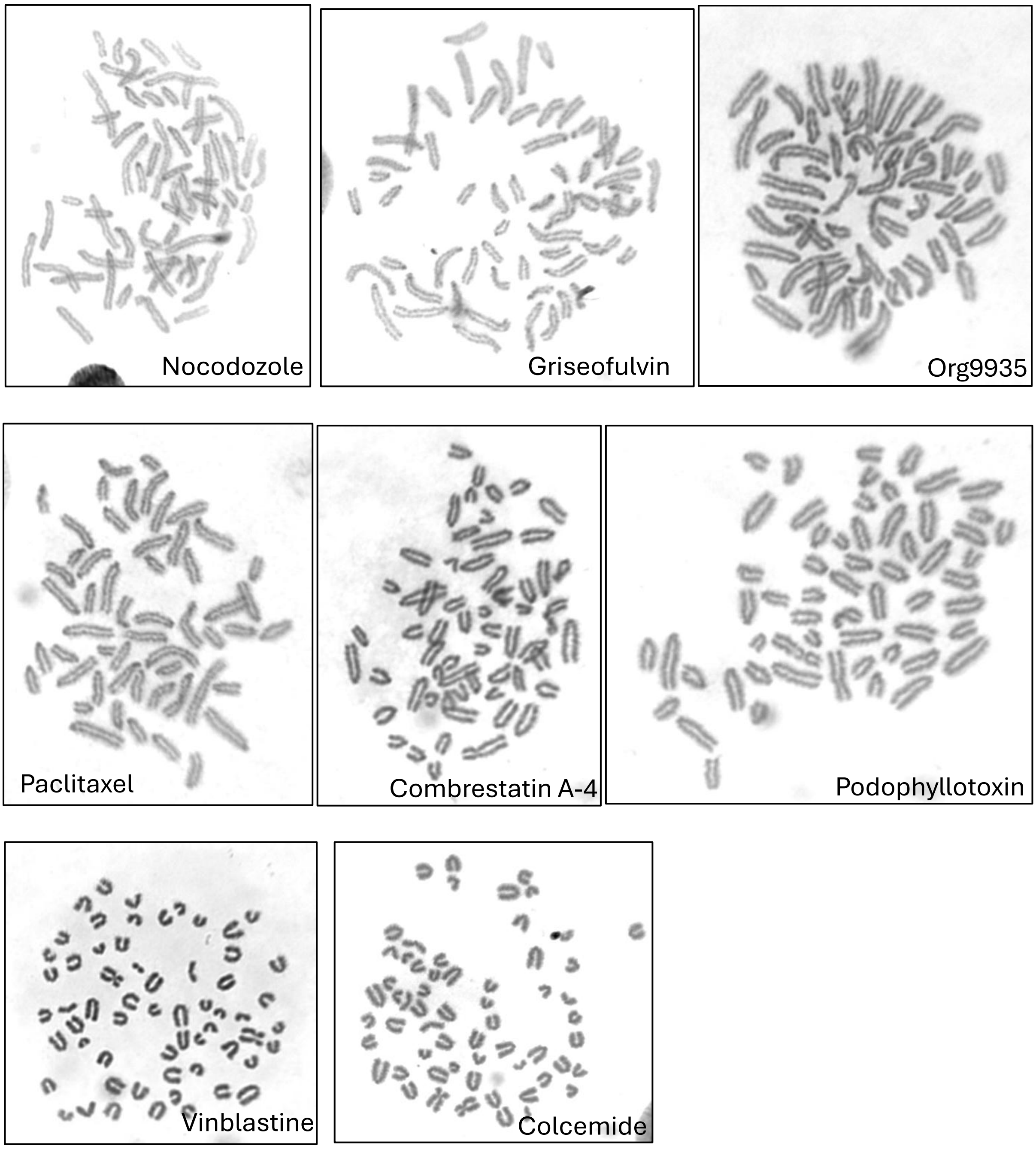

For the purpose of providing a representative example of the metaphases obtained for all tested compounds, the metaphases reported are all derived from the first experiment (90-minute exposure to colcemid.
